# Trouble and Repair in Child–Robot Interaction: A Study of Complex Interactions With a Robot Tutee in a Primary School Classroom

**DOI:** 10.3389/frobt.2020.00046

**Published:** 2020-04-09

**Authors:** Sofia Serholt, Lena Pareto, Sara Ekström, Sara Ljungblad

**Affiliations:** ^1^Division of Learning, Communication and IT, Department of Applied IT, University of Gothenburg, Gothenburg, Sweden; ^2^Division of Media and Design, School of Business, Economics and IT, University West, Trollhättan, Sweden; ^3^Division of Interaction Design, Department of Computer Science and Engineering, University of Gothenburg and Chalmers University of Technology, Gothenburg, Sweden

**Keywords:** child–robot interaction, education, social robotics, interaction trouble and repair, group interaction, robot tutee, in the wild, classroom study

## Abstract

Today, robots are studied and expected to be used in a range of social roles within classrooms. Yet, due to a number of limitations in social robots, robot interactions should be expected to occasionally suffer from troublesome situations and breakdowns. In this paper, we explore this issue by studying how children handle interaction trouble with a robot tutee in a classroom setting. The findings have implications not only for the design of robots, but also for evaluating their benefit in, and for, educational contexts. In this study, we conducted video analysis of children's group interactions with a robot tutee in a classroom setting, in order to explore the nature of these troubles in the wild. Within each group, children took turns acting as the primary interaction partner for the robot within the context of a mathematics game. Specifically, we examined what types of situations constitute trouble in these child–robot interactions, the strategies that individual children employ to cope with this trouble, as well as the strategies employed by other actors witnessing the trouble. By means of Interaction Analysis, we studied the video recordings of nine group interaction sessions (*n* = 33 children) in primary school grades 2 and 4. We found that sources of trouble related to the robot's social norm violations, which could be either active or passive. In terms of strategies, the children either persisted in their attempts at interacting with the robot by adapting their behavior in different ways, distanced themselves from the robot, or sought the help of present adults (i.e., a researcher in a teacher role, or an experimenter) or their peers (i.e., the child's classmates in each group). In terms of the witnessing actors, they addressed the trouble by providing guidance directed at the child interacting with the robot, or by intervening in the interaction. These findings reveal the unspoken rules by which children orient toward social robots, the complexities of child–robot interaction in the wild, and provide insights on children's perspectives and expectations of social robots in classroom contexts.

## Introduction

Over the past decades, research has explored the possibility of using social robots in a range of educational roles, including as teachers and tutors, peers, and novices (Belpaeme et al., 2018). For instance, the EMOTE project developed a robot with empathic qualities, which could tutor primary school students on tasks related to geography and sustainable development (Serholt and Barendregt, 2016; Obaid et al., 2018; Alves-Oliveira et al., 2019), the L2TOR project developed a robot that could tutor preschool children on second language learning (Vogt et al., 2019), whereas the CoWriter project developed a robot in the role of a novice, which children could teach handwriting skills to (El-Hamamsy et al., 2019). The motivations behind these efforts range from explorations of robots as technologies for supporting children's learning [e.g., language learning (Kory-Westlund and Breazeal, 2019)] and the development of targeted skills [e.g., self-regulated learning (Jones and Castellano, 2018)], to a conception of robots as solutions to various educational challenges, such as teachers' workload (Movellan et al., 2005) and a global teacher shortage (Edwards and Cheok, 2018). While social robots may not be used on a regular basis in education at present (Selwyn, 2019), researchers and developers continue to design novel applications for robots that aim to support education in various ways.

One caveat to this kind of research, and by extension to the benefit and usefulness of implementing social robots in education, lies in the fact that current robot solutions are expensive, have limited functionality, and are prone to breakdowns of both a social and technical nature. Ros et al. (2011) noted these difficulties during their extensive studies of Child–Robot Interaction (CRI) in a hospital setting; accordingly, they argued for the need to plan such studies appropriately by asserting that the robot used is mechanically robust, and by accounting for unpredictability in children's behavior. However, recent research suggests that these challenges are still prevalent (Belpaeme et al., 2018; Serholt, 2018), and this is partly related to the difficulty in predicting social behavior. As Honig and Oron-Gilad (2018) put it: “While substantial effort has been invested in making robots more reliable, experience demonstrates that robots operating in unstructured environments are often challenged by frequent failures” (p. 2). In CRI scenarios, social or technical breakdowns can lead to children's disappointment, loss of engagement (Ros et al., 2011), or even emotional distress (Serholt, 2018). An extended follow-up study showed that children also tend to remember such breakdown situations, even after 3 years (Serholt, 2019). However, little is known about the nature of these issues in CRI, and how children work to address or mitigate them in interaction. The lack of research on this topic provides a false presupposition that CRI is more frictionless than it actually is. By identifying and understanding the situations where robots fail in social interaction, it is possible to critically reflect on how to handle such situations from an educational and design perspective, while also furthering our understanding of how children interact with robots.

From the perspective of Interaction Analysis, breakdowns are usually preceded by what is known as “trouble” in interaction (Jordan and Henderson, 1995). Specifically, trouble in interaction becomes evident when it breaks the rhythmicity of an otherwise stable routine or interaction script, which is the given design in most CRI scenarios. When trouble occurs, people resort to repair strategies in order to handle the problem and avoid the occurrence of breakdowns (Jordan and Henderson, 1995). This process of trouble and repair probably becomes especially complicated when children deal with robots, since there is likely a mismatch between the children's and the robot's rules of interpretation—rules that are typically assumed to be somewhat aligned in everyday social interaction among people (Jordan and Henderson, 1995). In their systematic analysis of video data from five different Human–Robot Interaction (HRI) studies, Giuliani et al. (2015) found a number of differences in people's social responses to error situations in their interactions with robots. For instance, people displayed significantly more non-verbal social signals and spoke more when in a group or when an experimenter was present, vs. when they were alone. They also behaved differently depending on the type of failure, whether it was a social norm violation, i.e., “a deviation from the social script or the usage of the wrong social signals” (p. 3), or a technical failure. Giuliani et al. (2015) argue that evaluators of HRI systems should not discard data containing error situations, since it may contain valuable results.

As argued by Jordan and Henderson (1995), careful analysis of trouble in interaction “can often reveal the unspoken rules by which people organize their lives” and it is “one of the best methods for coming to an understanding of what the world looks like from somebody else's point of view” (p. 69). Hence, Interaction Analysis lends itself to exploring particular challenges related to designing robots for children, the expectations that children may have of interactions with robots, along with an understanding of the repair strategies children employ when their social expectations do not align with the social script of the robot. However, little is known about the detailed, sequential mechanisms by which interactions between children and robots play out in naturalistic settings such as classrooms, and the strategies that children employ in the face of trouble. In their recent literature review study, Honig and Oron-Gilad (2018) explored robot failures in HRI, including how people perceive and resolve these failures. However, the authors found that most such studies have been conducted in controlled, single-person environments, and that they therefore lack in ecological validity. Moreover, very few studies have considered children as the explicit target group. One exception is a previous experimental study where pairs of children aged 4–5 played a game with a robot that feigned getting lost, disobeyed the children's instructions, or made a mistake and recovered (Lemaignan et al., 2015). The authors were unable to affirm whether the children could perceive the difference between what they intended to be understood as a technical malfunction (i.e., the robot getting lost), and intentional social behavior (i.e., the robot disobeying the children, or making a mistake and recovering). The authors recommended that similar studies be replicated with older children. Another exception is an earlier study of interaction breakdowns between children and a robot tutor conducted by one of the authors of the current paper, where breakdowns were caused by both technical malfunctions, as well as social and pedagogical norm violations (Serholt, 2018).

Against this background, we present a qualitative analysis of video data obtained from a CRI field trial in a primary school classroom. As suggested by, e.g., Honig and Oron-Gilad (2018), the trial was designed to have high ecological validity, i.e., it took place in a familiar environment (the children's ordinary classrooms), and it included a variety of actors and artifacts. These actors and artifacts consisted of a social robot tutee seeking to learn arithmetic from the children, an interactive whiteboard displaying a mathematics game, groups of children in which one individual could interact directly with the robot at a time, a researcher in a teacher role, and an experimenter.

The initial aim of this field trial was to observe children's interactions with robots in naturalistic settings, in order to derive design recommendations for robot tutees. Yet, as we *familiarized ourselves with our data* (Braun and Clarke, 2006) through our qualitative, inductive approach, it became evident that the videos contained rich data regarding interaction trouble and repair strategies. Thus, the aim of the current paper is to explore trouble and repair in CRI. These findings do not only hold implications for the design of social robots for classrooms, but they also reveal the unspoken rules and/or silent expectations that children may have of robots in educational settings. The following research questions guide this study:

RQ1: What situations and/or behaviors constitute trouble in the child–robot interaction situation?
RQ2: What strategies do children employ when trouble occurs in the child–robot interaction situation?
RQ3: What strategies do the other actors (e.g., peer group members, researcher as teacher, and experimenter) employ when witnessing trouble in the child–robot interaction situation?

## Materials and Methods

We conducted field trials with a robot tutee under development in our research project Student Tutor and Robot Tutee (START), and an accompanying mathematics game at two primary schools in Sweden. The field trial constituted a first test of the children's interactions with the setup in a complex classroom setting with multiple actors. The trial took two full days at each school. The students participated in the trial in groups of four, scheduled by their teachers, and as part of their regular school activities.

### Apparatus

The technical setup consisted of the humanoid robot Pepper from Softbank Robotics^1^ and a digital mathematics game displayed on a wall-mounted screen. The mathematics game was adopted from a previously developed game called the Graphical Arithmetic Game, stemming from research on game-based learning and teachable virtual agents (Pareto, 2014). In our research project, the game has been updated and augmented to include a physical robot acting as a tutee and a co-player in the game (Pareto, 2017; Pareto et al., 2019). The use of teachable agents or robot tutees draws on learning-by-teaching and peer-assisted learning approaches, where children are engaged in the activity of teaching a novice or peer in order to further their own learning of a specific topic (see e.g., the CoWriter project: El-Hamamsy et al., 2019).

The mathematics game selected for this study constitutes a collaborative mini-game in the Graphical Arithmetic Game called 10-buddies. The game is a simple 2 player addition game, with the goal to add to ten by taking turns and choosing cards from the two players' respective card hands. Card values are graphically represented through colored blocks, ranging from values 1–9. In this case, a child and a fully autonomous robot tutee constitute the active players. However, as long as the robot tutee is at a novice stage, it does not actively play its own cards. Instead, the robot observes the child's choices and utilizes the existing question-and-answer repertoire of the earlier entirely text-based virtual agent, while it also exhibits socially interactive behaviors. This includes the display of some pre-programmed movement, gestures, and gazing behaviors, along with the implementation of a text-to-speech module in Swedish, in order to support verbal communication. The robot connects to the game through a local wireless network, and the game steers its behavior based on the child's actions in the game. In terms of the robot's verbal repertoire, there is a progression in what kind of questions the robot asks, depending on how well the children play and how well they manage to answer these questions. Typical questions in the beginning concern the overall game idea: how to score points and what the objects on the display mean. Then, the robot progresses to inquire about which cards will yield points, and finally, which cards are strategically smart to play considering future turns and possibilities. Hence, the robot consistently features as the children's inquisitive tutee whose goal is to learn how to play the game, and to improve its skills pertaining to mathematics. In the current study, each student group began with a novice robot tutee to teach, so all groups played both player turns, and answered the same type of inquisitive questions; the robot was programmed to ask such questions whenever the child selected a card from its hand. In order to facilitate progression in the interaction, the robot was designed to move on to the next step in the interaction if it did not receive, or was unable to perceive, any input from the game-playing child (such as a verbal response to its question). This occurred after a waiting period of 45 s, and was indicated by a verbal utterance, such as “Let's move on.” The robot could also return to an earlier question by asking, “Have you come up with the answer to this question: [*question*]?”

**Figure 1:**
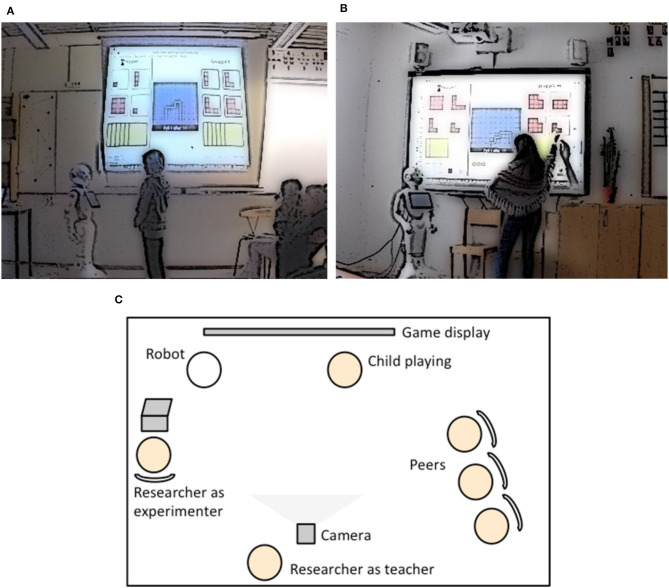
**(A)** Still image of the field trial setup at School A. **(B)** Still image of the field trial setup at School B. **(C)** Top-view illustration of the field trial setups.

The game and robot were placed in school spaces designated by the staff, meaning that the game had to be displayed on the schools' available equipment. In one school (School A), the trial was conducted in an empty classroom with a projector and wall-mounted canvas; in the other school (School B), a room for after-school activities with an interactive whiteboard was used. The game was displayed on the screen or the whiteboard with the robot standing in front of the display together with the game-playing child. The children in each group took turns playing the game with the robot; the children currently not playing were seated next to the scene, accompanied by one of the authors (researcher in teacher role), who is also a licensed teacher with 15 years of teaching experience. Her role was to facilitate and organize the children's collaboration during the sessions, observe the interaction, and manage a video recorder. Another author (the experimenter) was tasked with handling the technical aspects of the game, i.e., starting the game and making sure that everything was working, while also executing the children's choices of cards during their turns in School A where the display was not interactive. Finally, a video camera placed in the middle of each room captured the game display, the robot, and the child from behind. For illustrations of the interaction sessions and field trial setups, see Figure 1.

### Participants

Two classes from each school participated in the study: two 2nd grade classes from School A and two 4th grade classes from School B, i.e., children of ages 8 and 10 years old. The classes were selected based on active interest from the children's respective teachers to enroll in our research project. Thus, the same children participated in a workshop on a previous occasion, which consisted of a robot-programming task and a post-workshop questionnaire (Pareto et al., 2019). The children had never played the Graphical Arithmetic Game before. In total 69 children across 19 groups participated in the trial: 28 children from the 2nd grade, and 41 children from the 4th grade. In the current study, we randomly sampled the sessions of nine groups for analysis (*n*_children_ = 33; 15 female, 18 male; 17 second-graders, and 16 fourth-graders).

### Procedure

Prior to the study, the class teachers divided the children into groups of three or four. During the study, the teachers excused each group from their classroom to take part in the trial in the allocated room for 30 min each. The trial sessions then proceeded as follows: First, the researchers welcomed the children, described the aim of the study, and asked for confirmation of the children's previous assent to participate in the study. Second, the researchers briefly explained the game's aim and rules. They also explained that the children would take turns playing with the robot, but that the group members were encouraged to help the child playing the game, and to suggest answers to the robot's questions. Then, a video recorder was activated, and the game session was initiated. During the session, the children took turns on a voluntary basis to play the game together with the robot, where each child was allowed to play for about 6–7 min before being asked to take a seat, whereupon another child could volunteer. In the analyzed sessions, the children played on average 10 cards each, including the choices they made for the robot. After 30 min, the researchers thanked the groups for their participation, held a debriefing session about their experience, and followed them back to class.

### Data Collection and Analysis

During the field trial, we collected video recordings of the interaction sessions. As mentioned previously, nine of the interaction videos were randomly selected for analysis, which amounted to a total of 3 h and 15 min of video data. For this study, we adopted a qualitative inductive approach, drawing on thematic analysis (Braun and Clarke, 2006) and Interaction Analysis (Jordan and Henderson, 1995).

### Thematic and Interaction Analysis

The first phase in our analysis involved what Braun and Clarke (2006) refer to as *familiarizing yourself with your data*, i.e., the search for interesting areas of study in the material without explicit protocol. Specifically, the videos were viewed independently by two of the authors (henceforth referred to as coders), who made notes regarding their observations. These observations were discussed with the remaining authors through a joint data session where all authors viewed and discussed the content of selected videos. At this stage, consensus was reached that the data contained rich material regarding interaction trouble and repair strategies.

The next phase involved conducting interaction analyses of the sessions (Jordan and Henderson, 1995). First, the videos were divided between the two coders who each created interaction transcripts for half of the videos. The interaction transcripts contained high-level documentation of the sequential interaction processes, i.e., what each actor in the material was doing at specific times, descriptions of the interaction between the different actors, along with the coders' analyses of the interactions. One such transcript was produced for each game-playing child in their respective group (*M*_duration_ = 5 min 55 sec; *min* = 1 min 38 s; *max* = 10 min 45 s), meaning that between three and four transcripts were produced for each interaction session (33 transcripts in total). Segments containing trouble in interaction were documented in detail, whereas segments containing fluent turn-taking and gameplay were just commented as such. To identify trouble, any situation, which seemingly disrupted the interaction flow, was considered.

The third phase in the analysis consisted of coding the data, in which the two coders independently coded half of the videos each. We followed the three-stage process for coding qualitative data suggested by Campbell et al. (2013): (1) developing a coding scheme with as high intercoder reliability as possible based on a sample of transcripts (typically 10%), (2) negotiating coding disagreements among coders until reaching acceptable levels of intercoder agreement (as the recommended approach in exploratory research), and (3) deploying the coding scheme to the full set of transcripts. During the first stage, a preliminary coding scheme was developed by the more experienced coder on a sample of two sessions, which was tested by the other coder under supervision. This procedure generated a list of codes, which each contained a qualitative description of an observed action along with a label. The codes were organized into inductively formulated code families, each denoting a common topic (Campbell et al., 2013). Four transcripts out of 33 (12%) were coded by both coders independently and checked for intercoder reliability. Following Campbell et al. (2013), intercoder reliability was calculated as the number of common instances of codes (i.e., agreement in coding) divided by the total instances of codes (i.e., agreement + disagreement). The average level of intercoder reliability was 73%. For the second stage, the coding disagreements (33 out of 125 codes) were analyzed and discussed by the coders. The most frequent differences were whether subtle non-verbal actions occurred or not (18 disagreements), and whether the child or the adult initiated a help action (7 disagreements). The coding scheme was refined to address these differences. For the third stage, the remaining sessions were divided between the two coders and coded independently. Given our inductive approach, the list of codes evolved and was continuously discussed, compared, and unified during the process, producing a joint coding scheme. The final coding scheme consists of seven (primary) code families and 36 (secondary) codes (see Supplementary Materials Table A).

The final phase in our analysis involved developing themes to describe the nature of trouble in CRI, and the following repair strategies. This phase was carried out by one of the authors who developed themes based on the coding scheme and interaction transcripts. The themes were discussed and reformulated through several iterations with the remaining authors.

## Results

In this study, we set out to explore trouble and repair in CRI. This analytical interest stemmed from our observations of children's group interactions with a robot tutee in a classroom setting, wherein trouble (and repair) seemed prevalent. By means of Interaction Analysis and thematic analysis, we explored situations of trouble and repair, which constituted 26.4% of the sampled video corpus (the remaining segments of the video corpus depicted what we considered to be fluent interactions).

In this section, we present our findings in individual subsections for each research question. Within each subsection, main themes are represented through italicized, bold, font (i.e., ***main theme***), subthemes are indicated as such through bold font (i.e., **subtheme**), and translated excerpts derived from the Interaction Analysis are shown for illustration and discussion purposes. Individual children are denoted through their participant IDs (**C** for game-playing child accompanied by a number). Table 1 provides an overview of all themes and subthemes.

**Table 1:** Overview of themes and subthemes derived from the thematic analysis.

**Research question**	**Main themes**	**Subthemes**
RQ1: Sources of trouble	Active social norm violations	Makes irrelevant comments
		Interrupts Signals dismissal through non-verbal behavior
	Passive social norm violations	Fails to act at its designated turn in the game
		Fails to respond verbally
RQ2: Children's repair strategies	Adapt to the robot	Exaggerate articulateness
		Modifying tutoring approach
		Seeking to understand interaction form
	Establish distance to the robot	Making the robot invisible
		Give up
	Shift focus to human actors	Seek affirmation
		Request help
RQ3: Strategies of other actors	Offer help	Provide guidance to the child Intervene (or interfere) in the interaction

### The Sources of Trouble

We found that situations and/or behaviors that constituted trouble in this particular CRI situation (i.e., the sources of trouble) were related to the robot's social norm violations, which were either ***active social norm violations*** (41%) or ***passive social norm violations*** (59%). Although these violations could be traced back to technical issues or limitations with the robot, this analysis is concerned with exploring these situations from an interaction perspective. Hence, sources of trouble are considered from the perspective of how it might be interpreted in social interaction.

Trouble stemming from the robot's ***active social norm violations*** manifested in different ways. Yet, the commonality was that these behaviors were unexpected and undesirable. First, the robot sometimes **made irrelevant comments**, which constituted 44% of all active social norm violations. For example, when C3 was in the process of explaining to the robot that they needed to try again, the robot responded with the following contextually irrelevant comment:“*Yes I know that 7* + *3* = *10.”* Second, the robot sometimes **interrupted** (33%) the child speaking. For instance, as C1 was in the process of providing a response to one of the robot's questions, the robot unexpectedly announced, “*Now we continue to play!”* which could be perceived as general disinterest or disregard for what the child had to say. Third, trouble also surfaced when the robot **signaled dismissal through non-verbal behavior** (23%), e.g., when it turned its back to the child in the middle of an interaction. In one situation, the child called out to the robot in order to encourage it to turn around and face him/her; instead of doing so, the robot merely responded: “*Yes, I hear you”* which, in interactions between humans, would likely be interpreted as disengaged and dismissive behavior.

Regarding the robot's ***passive social norm violations***, there were several situations when the robot simply failed to act as expected. For instance, the robot could lose its connection to the game and consequently **fail to act at its designated turn in the game**, yet this only accounted for 10% of its passive behavior. More common was the robot's **failure to respond verbally** (90%) to the child when such behavior seemed mandated. This could occur during the child's attempts to greet the robot, but also during dialogues connected to the gameplay. For instance, for C4, the robot inquired as to how they would receive points in the game, for which he provided a verbal explanation; the robot, however, only acknowledged his explanation non-verbally (by nodding), which caused trouble since he became uncertain as to whether the robot had actually understood.

In Table 2, data regarding the number of times each source of trouble was observed, along with the number of children across the whole dataset who encountered it, is presented. In total 32 of the 33 children in this study encountered some form of trouble during their sessions.

**Table 2:** Number of occurrences and children who encountered each source of trouble.

**Main themes and subthemes**	**No. of occurrences**	**Percentage of children who experienced each theme**
Active social norm violation	64	88%
Makes irrelevant comments	28	55%
Interrupts	21	42%
Signals dismissal through non-verbal behavior	15	18%
Passive social norm violations	92	73%
Fails to respond verbally	83	70%
Fails to act at its designated turn in the game	9	18%

*For main themes, child percentages are based on the whole sample (n = 33); for subthemes, child percentages are instead based on the number of children within each main theme*.

### Children's Repair Strategies

Our analysis shows that children use different repair strategies in different situations. Specifically, the children either persisted in their attempts at interacting with the robot by modifying their behavior and ***adapting to the robot*** in different ways, ***distancing themselves from the robot***, or ***shifting focus to the human actors*** present (i.e., the researcher in a teacher role, the experimenter, or the child's peer group members). These main themes will be presented in turn below. Notwithstanding, the same child typically employed a variety of strategies within the same session, such that these categories are not by any means mutually exclusive on a child-by-child basis.

Regarding children's methods for ***adapting to the robot***, the children could **exaggerate articulateness**. In such cases, children could change the ways in which they communicated verbally with the robot by either shortening their responses to simple keywords, or strengthening the volume and clarity of their verbal communication. In the excerpt shown in Table 3, C2 is playing the game, whereby the robot poses a question.

**Table 3:** Excerpt for C2.

**C2**		**Male, 2nd grade, school A, second player of his group**		
**Time**	**Actor**	**Verbal**	**Non-verbal**	**Note**
18.22	Robot	Why are there 10 squares in the enclosed area?		
18.25	Child	I don't know.		
	Robot		Nods	Trouble: fails to respond verbally
18.34	Child	Listen. I don't know	Moves closer to the robot and speaks close to its face	Repair: exaggerate articulateness

As can be observed in this example, the robot poses an inquisitive question regarding the game board to gauge the significance of the value ten. C2 responds verbally that he does not know why there are ten empty boxes in the enclosed area on the game board. That the robot nods but fails to respond verbally indicates to C2 that the robot has not properly heard or understood his answer, causing temporary trouble. In response, C2 attempts to repair this trouble by trying to get the robot's attention (when he moves closer and says: “*Listen”*), and by trying to make his response audible.

In contrast to these exaggeration strategies, we also found that the children tried to adapt to the robot in more social ways, e.g., by **modifying their tutoring approach**. Specifically, this could entail the children elaborating upon a mathematical concept, or explaining in a different way than they had done initially. In some cases, these modified explanations were complemented by visual demonstration on the game display through a variety of gestures, such as pointing to elements in the game. The children could also ask the robot to repeat or explain itself (e.g., “*I didn't hear what you said in the beginning”* [C26]), or simply instruct the robot on which cards to play. Taken together, such adaptations could indicate that the children perceived the robot as a social other capable of perceiving and interpreting complex human reasoning. In the excerpt shown in Table 4, the robot asks C4 a question while he is playing the game.

**Table 4:** Excerpt for C4.

**C4**		**Male, 2nd grade, school A, first player of his group**		
**Time**	**Actor**	**Verbal**	**Non-verbal**	**Note**
10.22	Robot	Have you come up with the answer to this question: how do we get points?		The first part of the question denotes that the robot has asked this question before, but not perceived a response
10.28	Child	We will fill these boxes.	Points to the enclosed area of the game board	Repair: modifying tutoring approach
	Robot		Nods	Trouble: fails to respond verbally
10.33	Child	Then we get stars. There are points.	Points to the score meter	Repair: modifying tutoring approach

Earlier in the interaction, the robot had already asked how they receive points, but had not perceived or understood the response. Against this experience, C4 thus tries to modify his response by complementing his verbal explanation with gestures directed at the game board. In response, the robot simply nods and fails to respond verbally, which is interpreted by C4 as a signal that the robot does not quite understand. Hence, C4 once again tries to modify his tutoring approach by explaining the game mechanics in a different way (with reference to the scoring of points by acquiring stars).

Another way in which children tried to adapt to the robot concerned their **seeking to understand the interaction form**, where they also seemed open toward interacting on the robot's terms. While this was also the case when the children exaggerated their articulateness, this subtheme differed in relation to the children's seeming curiosity. For instance, they could increase their proximity to the robot and perform exaggerated gestures in an attempt to make the robot perceive and recognize their interaction endeavor. Yet, unlike the situations where the children would only utter keywords, presumably for the sake of the robot's speech recognition difficulties, these communication attempts seemed more related to the children's desire to establish communication with the robot (e.g., C33 who asked the robot, “*What are you doing?”* when it failed to respond). In some cases, the children would wait patiently for the robot to act while they stood in front of it. In other cases, the children would mirror the robot's non-verbal behavior (e.g., C25, who switched from verbal communication to mirroring the robot's frequent head nodding).

In contrast to adaptive behaviors, the children also responded to trouble in interaction by ***establishing distance to the robot***. This strategy mainly occurred after a long sequence of trouble; hence, it was usually preceded by some form of overt expression of emotional distress such as discomfort or irritation. Some of the children established distance by **making the robot invisible**, i.e., a form of domination technique. Specifically, this could manifest itself through the children talking over the robot, i.e., speaking simultaneously as the robot, but not directed at the robot as such. Some children simply ignored the robot's questions completely, whereas some children took a less overt approach and acknowledged the robot's questions, but provided an indifferent response (e.g., “*Mm”*). They could also interrupt the robot by quickly answering “*Yes”* or “*No”* at the start of the robot's utterance. Some children also chose to **give up** on the interaction, either by walking away and taking a seat, or by stating that they did not want to continue the interaction, as demonstrated in the following excerpt (see Table 5).

**Table 5:** Excerpt for C7.

**C7**		**Female, 2nd grade, school A, fourth player of her group**		
**Time**	**Actor**	**Verbal**	**Non-verbal**	**Note**
25.44	Robot	Okay, 5.		
25.50	Child	Yes	Leaning forward	Repair: exaggerate articulateness
	Child		Standing still	Repair: seeking to understand interaction form
25.55	Robot	Now we have 4 points.		
26,01	Child	I don't want to play anymore.		Give up

Finally, children's strategies consisted of ***shifting focus to the human actors*** who were present (either the researchers or their peer group members). This typically occurred when the children had exhausted other repair strategies more directed toward the robot. From these other actors, the children often **sought affirmation** regarding their responses to the robot's dialogue (e.g., checking with researchers or peers that their particular response would be appropriate), but also related to gameplay choices (e.g., asking peers or researchers to confirm that their card selection would afford points). In more difficult situations, however, the children would **request help** in open-ended and explicit ways, indicating both verbally and non-verbally that they did not understand how to proceed in the dialogue with the robot or the game.

### Strategies of Other Actors

**Figure 2:**
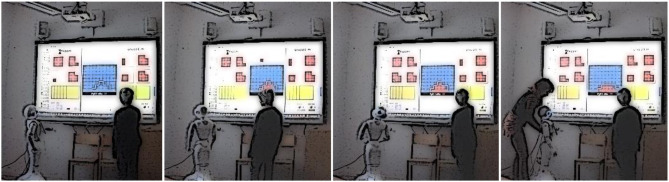
Still images from an interaction session illustrating the experimenter intervening when the robot has turned its back to the child.

In terms of the other actors present in the interaction sessions (i.e., the child's group members, the researcher as experimenter, and the researcher in a teacher role), their repair strategies consisted of ***offering help*** in various ways. In most cases, they tried to **provide guidance to the child**, which meant that they addressed the game-playing child directly, and conveyed various forms of scaffolding for interacting with the robot successfully, but also regarding strategic moves in the game. They also **intervened (or interfered) in the interaction** by responding to the robot directly. For instance, the peers could call out the correct answer, or try to get the robot's attention, but this was quite rare. On a few occasions when the robot signaled dismissal through non-verbal behavior by turning its back to the children, the experimenter intervened as shown in Figure 2.

In terms of providing guidance to the child, the experimenter, who possessed technical knowledge about the robot and the game, could suggest specific verbal formulations that the robot would understand. The peer group members could also become involved in this process of trouble and repair, leading to a complex interaction situation. Often times, the peers drew on their previous experiences having heard similar questions from the robot during their turns. The excerpt in Table 6 provides an illustration of when the experimenter and the peer group members provided guidance to C15. Right before the excerpt, C15 indicates that he does not know how to answer the robot's question and consequently stays silent while grabbing the robot's hand.

**Table 6:** Excerpt for C15.

**C15**		**Male, 2nd grade, school A, second player of his group**		
**Time**	**Actor**	**Verbal**	**Non-verbal**	**Note**
14.48	Experimenter	It is your job to answer Pepper [the robot].		Repair: provides guidance to the child
14.51	Child		Releases the robot's hand and continues to play	
14.55	Experimenter	Ask your peers what they said.		
14.59	Peers	That you should get 10.		Repair: provide guidance to the child
15.00	Child	To get 10.	Talks close to the robot	Repair: exaggerate articulateness
	Robot		Nods	Trouble: fails to respond verbally
15.03	Experimenter	One more time.		Repair: provides guidance to the child
15.08	Child	To get 10.	Talks even closer to the robot	Repair: exaggerate articulateness
	Robot		Gazes at the game	Trouble: fails to respond verbally
15.11	Experimenter	Stand in front of it so Pepper [the robot] sees you.		Repair: provides guidance to the child
15.16	Child	(laughingly) To get 10.	Standing on his toes right in front of the robot	Repair: exaggerate articulateness
15.23	Robot	Let's leave this [question] now.	Gazes at the game	Trouble: makes irrelevant comment
15.25	Peers		Laughing	

As can be observed in this excerpt, the experimenter tries to guide the child when she notes that the child does not know how to answer the question. She does so by involving C15's peer group members so that they can provide input on what to say to the robot based on their experience from the first player round. What follows is a series of trouble and repair strategies consisting of the robot failing to respond verbally, and C15 trying to make himself understood through exaggerated articulateness. These attempts go unsuccessful, and the interaction ends with the robot proclaiming that they should give up on that question and proceed instead.

## Discussion

This qualitative study has explored interaction trouble and repair in the field of CRI. Our analytical interest came about through our initial observations of children's classroom interactions with a robot tutee in the context of a collaborative game in mathematics, which took place in groups of children who took turns actively playing the game and teaching the robot.

Our research questions concerned what situations and/or behaviors constitute trouble in such a CRI setting, what repair strategies children employ to address interaction trouble, and how onlookers (researchers and peer group members) respond when witnessing the trouble. The results indicated that the primary source of interaction trouble related to the robot's social norm violations. Notwithstanding, there were a few additional situations, not due to the robot, which caused trouble as well. For instance, in one group in particular, the peer group members not actively playing the game with the robot disrupted the interaction by shouting out various directives at the game-playing child. Whereas, one of the children was able to ignore this behavior during her turn, another child became very distracted and began jumping around the room and throwing himself on the floor in an attempt to entertain his peers. However, for the sake of limiting our scope, we omitted such (rare) cases from further analysis.

Regarding the robot's social norm violations (RQ1), one could argue that these were, in a way, always a result of technical issues rather than an intentional design choice. Yet, the (social) interactional setting did not provide any actual opportunities for children to differentiate between social vs. technical issues, making it futile to discuss these differences from an interaction perspective. Our findings resonate with an earlier literature review on failure in HRI (Honig and Oron-Gilad, 2018). Indeed, all sources of trouble identified in the current study have been observed in HRI before, albeit with the obvious contextual variations. Compare, e.g., the similarities between our subthemes and the descriptions of errors and symptoms identified by Honig and Oron-Gilad (2018): **Interrupts** vs. “timing speech improperly,” **makes irrelevant comments** vs. “producing inappropriate speech or erroneous instruction,” **fails to act at its designated turn in the game** and **fails to respond verbally** vs. “producing no action or speech (irresponsiveness),” and finally, **signals dismissal through non-verbal behavior** vs. “producing unexpected or erratic behavior.” It thus seems that these issues are not limited to a specific set of robot products, but actually a common challenge faced by several research projects in HRI; examples of this are, however, much rarer in CRI.

Turning to children's repair strategies (RQ2), these were many and varied in this study, including adapting to the robot's shortcomings in perception by exaggerating articulateness, adapting to its lack of knowledge in mathematics by modifying their tutoring approach, or by adapting to what they believed to be the robot's interaction modalities. We found that children used these strategies not only in response to the trouble currently taking place, but also as proactive measures throughout the interaction sessions. This suggests that children reiterated their understanding of the robot's capability as the interaction progressed. Children also shifted their focus to the human actors in the room, and sought their guidance with the interaction and task. They could also establish distance to the robot in various ways. Moreover, children used different strategies in close succession in a trial-and-error fashion. For instance, they could begin by modifying their tutoring approach, and then decide to request help from peers or researchers, and finally end up giving up on the interaction altogether.

As our analysis of the strategies of the peer group members and the researchers reveal (RQ3), the children did not necessarily need to request help as this was in many cases offered voluntarily. Typically, such guidance consisted of scaffolding the child currently interacting with the robot on what to say, and how to say it, in order for it to be perceptible to the robot. In other cases, peers and researchers intervened and spoke directly to the robot (or when the experimenter needed to physically turn the robot around to face the child); this type of intervention was, however, quite rare in our video data.

Taken together, the presence of additional actors in the room made various forms of support possible during the interaction. In contrast to one of the author's earlier studies of breakdowns in CRI (Serholt, 2018), children in the current study were perhaps able to avoid breakdowns largely due to the presence of other actors (researchers in particular), which enabled a form of collaborative repair work to take place. Indeed, children often turned to their peers and the researchers to repair troublesome situations. According to Serholt (2018), collaborations among peers during CRI can allow for a higher level of social support. However, it can also have certain drawbacks for the learning situation, such as children ignoring or mocking the robot that is supposed to facilitate their learning processes. Similar tendencies were found in the current study, specifically in relation to our subtheme **making the robot invisible**. While we did not observe mocking behaviors toward the robot *per se*, it is likely that the presence of adults (the researchers) actually discouraged children from such overt expressions of discontent. This should be considered from the wider perspective of implementing social robots in classrooms, where allowing children to interact with a robot on their own or in groups, vs. only in the presence of their teachers, requires understanding of the tradeoffs in order to reach a conscious and sensible solution. At present, research implies that children should not be left alone with educational social robots at all (Serholt et al., 2017; Newton and Newton, 2019).

Social robots are typically autonomous, embodied robots that may vary in form and behavior, but that are developed to follow certain social behaviors that is expected in its role. A previous study showed that people cooperated more with a robot whose social behavior was matched appropriately with a task (Goetz et al., 2003). This suggests that the willingness of children to collaborate with social robots in the classroom may depend on the extent to which its behavior fits the task and the overall situation. Another aspect concerns what kind of mental processes a robot in the classroom may facilitate when collaborating with children. An important related field to understand aspects of human cognition in HRI are social and cognitive neuroscience studies of human–robot and human–human interaction (Cross et al., 2019). For example, Rauchbauer et al. (2019) used functional magnetic resonance imaging (fMRI) to investigate neurological differences when people carried out a conversation with a robot compared to a person as an interaction partner in a task. The brain imaging findings revealed that human interaction led to engagement of brain regions associated with higher-order social cognitive processes, including the temporo-parietal junction. Performing the same task with a robotic interaction partner instead activated dorsal frontal and parietal brain regions. This indicates that human interactions engage more social motivation and mentalizing processes, while interactions with robots recruit additional executive and perceptual resources. This reveals some of the limitations of interactions with robots, and points toward the importance of peers and teachers to stimulate higher-order social cognitive processes among children.

From a design perspective, there are many ways in which these results can be considered and used. As suggested in previous work, robot interaction design may benefit from including socially based recovery strategies following a breakdown or trouble in interaction in order to promote long-term acceptance. Although we have studied a robot tutee only, we believe that our findings can be valuable for the development of social robots for children in general. Indeed, the social aspects of interaction with robots is not specific to the tutee role, even if, say, children's perceptions of the robot as a novice may have made them more forgiving toward its misunderstandings. According to Uchida et al. (2019), HRI researchers should not only focus on improving a robot's dialogue capability, but also consider ways to encourage cooperative intentions from users so that the user and robot will adopt an equal share of responsibility for breakdowns in dialogue. This is, indeed, interesting, and perhaps quite relevant for robot tutees, since much responsibility for joint understanding should probably fall on the tutor (child) rather than the tutee. We can already see this taking place in some of the children's adaptive strategies, specifically when they **modified their tutoring approach** in different ways. Of course, for this to be potentially beneficial, it would require the robot to be perceptive to these strategies.

Currently, off-the-shelf social robots are rather expensive, and extremely limited in functionality. Using a social robot in a classroom also requires technical expertise; not to mention the maintenance and updates required. For instance, when Davison et al. (2020) recently deployed a social robot in a classroom for 4 months in an unsupervised study, the researchers conducted all maintenance at particular times after school hours, meaning that there were times during the school day(s) when the robot could not be used as planned. It is paramount to apply and evaluate novel CRI systems, but considering the current technical limitations, these are far from ready to be implemented in schools to support teaching on a large scale. Conversational systems lack understanding of meaning and context and typically act on scripts in a pre-designed type of conversation (Serholt, 2018). Although there are noteworthy examples of somewhat long-term studies of autonomous social robots being conducted in classrooms (Serholt and Barendregt, 2016; Alves-Oliveira et al., 2019; Davison et al., 2020), many studies still require some degree of teleoperation for the interaction to work smoothly, particularly when it comes to verbal communication (Kory-Westlund and Breazeal, 2019; Vogt et al., 2019). This suggests that interaction trouble will likely continue to be a prominent feature of conversational interactions with autonomous social robots. It is our conviction that children cannot be expected to possess the skills necessary to repair all troublesome situations that follow, especially since educational robots can only be used in such delimited contexts (educational robots are seldom designed to function within more than a few specific educational activities), and duration during an ordinary school day. From this perspective, it is the responsibility of the designers of robots to make sure that the interaction works somewhat fluently. From a wider perspective of social robots in education, it is also necessary to consider the ethical aspects of implementing them in schools (Sharkey, 2016; Serholt et al., 2017).

### Limitations and Future Directions

There are several limitations to this study that should be considered. First, the study was limited to two schools in Sweden, the sample size was rather small, while we only considered a particular CRI setup. This makes our findings difficult to generalize to other contexts. Nevertheless, social interaction with robots, and children's expectations of such interactions discernible through their repair strategies, constitutes a first step in understanding these issues more generally. This study did not focus on the children's experiences of the interaction, their views of social norms, or their preferences in teaching methods and learning experiences. We welcome future research that can demonstrate additional themes to explain trouble and repair, as well as other entryways to this topic relevant for CRI.

Second, the children in our study had some previous experience of programming the robot to execute simple dialogues. This could have influenced their perceptions of the robot as a machine with a limited social repertoire. Future research could potentially do a comparative study of how children handle trouble and repair depending on their levels of previous experience with robots.

Third, the interaction sessions analyzed in this study were quite brief and short-term. This means that the interactions were likely affected by a certain novelty effect, and that children's repair strategies could be developed even further after some time. The next step would be to investigate if the robot is perceived to add stress to the learning situation and how it is perceived during long-term use. Generally speaking, future research should continue working toward making HRI and CRI studies more long-term.

Fourth, another influential factor not yet touched upon in this paper relates to the mathematics game. Although our study was mainly concerned with exploring the social interaction between children and robots, the interactive display held a mediating role throughout the interactions. It constituted a boundary object for the children and robot to interact around, which conveyed awareness of the social situation they shared (e.g., the robot knew what cards the children played and commented on their actions). Hence, the task was not purely verbal; it was also graphically represented on the game board, around which the children and robot had a joint task. Future research should explore the influence of such boundary objects in CRI.

Fifth, a methodological limitation to this study is its lack of validation of the intercoder reliability level after the coding scheme was developed; instead, we relied on continuous intercoder discussions and agreements to address reliability. Although the aim of this study has been to provide a theoretical account of trouble and repair in CRI, some quantitative results are also presented in relation to the theme *sources of trouble*; thus, these findings should be interpreted with care.

Finally, although we strived toward making this study naturalistic and ecologically valid, it was not feasible to include the children's actual teachers in the study due to the need for technical expertise in operating the system. It is possible that regular teachers without any experience in robotics would employ other strategies for supporting the children than the researchers did, which should be considered in relation to our results. The study of teacher repair in CRI could be an interesting avenue for future research, which we intend to explore once our robot design has reached a more developed stage, also incorporating and evaluating a set of repair strategies in the face of trouble. Furthermore, it would be interesting to explore the connections between certain forms of trouble and certain forms of repair strategies. Due to the explorative nature and relative small-scale of this study, however, this was not possible to do here.

## Conclusion

In this paper, we have explored trouble and repair strategies in children's interactions with a robot tutee in an educational setting. The aim of this study has been to shed light on the interaction issues in CRI under the premise that such issues can never be completely avoided or designed away. Trouble and repair in social interaction, while highly contextual, is also universal. Children make use of the strategies that they already know from human communication, but our study further demonstrates that having robots as social interaction partners introduces additional layers to the interaction. This makes this research, and similar future studies in this area, an important contribution not only to the design and evaluation of educational robots, but also for furthering our understanding of what it means for children to interact with and develop relationships with social robots.

## Data Availability Statement

The datasets generated for this study will not be made publicly available because video recordings contain personal identifiable information. Requests to access the datasets should be directed to Lena Pareto, lena.pareto@hv.se.

## Ethics Statement

This study was carried out in accordance with the recommendations of CODEX—Rules and Guidelines for Research established by the Swedish Research Council. The protocol did not require ethical approval according to Swedish law as no sensitive personal information about the subjects was collected. All child subjects' parents/legal guardians gave written informed consent, and all child subjects gave written assent, as well as verbal assent at the start of each session, in accordance with the Declaration of Helsinki.

## Author Contributions

SE and LP carried out the study and collected the empirical data, created the interaction transcripts, and coded the video data under the guidance of SS and SL. SS conducted the thematic analysis under the guidance of LP and SE, drafted the manuscript, and held the overall responsibility for bringing individual sections together. All authors contributed to the authoring of the final manuscript and contributed to the research design.

### Conflict of Interest

The authors declare that the research was conducted in the absence of any commercial or financial relationships that could be construed as a potential conflict of interest.

## Footnotes

^1^https://www.softbankrobotics.com/emea/en/pepper
