# Supplementary material for: Trouble and Repair in Child–Robot Interaction: A Study of Complex Interactions With a Robot Tutee in a Primary School Classroom
Source: Front Robot AI. 2020 Apr 9;7:46. doi: 10.3389/frobt.2020.00046 (PMC7805844; doi:10.3389/frobt.2020.00046)
Supplement: Supplementary file 1 [file Data_Sheet_1.PDF]

Supplementary Material

1 Coding Scheme

The coding scheme presented in Table A was developed inductively based on video analysis of children's interactions with a robot tutee in an educational setting. The codes included in the scheme focus on trouble and repair in communication.

Table A. Coding scheme

**Game-playing child's response to non-fluent/disruptive interaction**			
**Category**	**Label**	**Code**	**Qualitative description**
**Interaction towards robot**	#uh	repeats (louder, articulated)	*when the child repeats the same answer again, typically louder and/or more articulated*
	#st	responds clearly (close)	*when the child answers Pepper directly with clear pronunciation, typically using single keywords or short phrases*
	#sa	responds differently	*when the child changes strategy and responds using different words*
	#vkp	chooses card (through) Pepper	*when the child tells Pepper which card to play although experimenter selects card*
	#sp	responds by pointing	*when the child explains to Pepper by pointing and gesturing at the game board*
	#hif	doesn't hear the question	*when the child asks, "what did you say?" (to Pepper), or, "what did it say?" (to adults) – wants Pepper to repeat*
	#ap	interrupts Pepper when it talks or poses question	*when the child starts talking to Pepper about something else while Pepper is talking or posing a question*
	#skp	seeks contact (with) Pepper	*when the child clearly tries to get contact with Pepper (moves in front of, yells at Pepper, calls out)*

	#hp	mimics Pepper	*when the child mimics Peppers behavior (e.g., nods, gestures)*
	#avv	bides time	*when the child just stands in front of Pepper and does nothing*
**Distancing**	%ip	ignores Pepper	*when the child does not listen to Pepper although it asks a question*
	%so	responds with disinterest	*when the child barely acknowledges Pepper's question/comment and proceeds with what (s)he is doing*
	%ob	discomfort	*when the child seems uncomfortable, e.g., flaps with arms, pulls at his/her clothing, etc.*
	%irr	irritated	*when the child expresses frustration (e.g., seems irritated, mutters, or paces in a circle)*
	%va	wants/chooses to quit	*when the child walks away from the game and Pepper voluntarily or asks to quit*
**Adult-directed**	>this	requests help with game	*when the child clearly turns to the adults and explicitly requests help verbally or nonverbally about how the game works*
	>thip	requests help with Pepper	*when the child clearly turns to the adults and explicitly requests help verbally or nonverbally about how to respond to Pepper*
	>bis	seeks confirmation regarding game	*when the child already has a suggestion for a card but seeks confirmation from adults before making the selection*
	>bip	seeks confirmation regarding dialogue with Pepper	*when the child already has an answer for Pepper but seeks requests confirmation from adults before saying the answer*
**Peer-directed**	&thks	requests help with game	*when the child clearly turns to peers and explicitly requests help verbally or nonverbally about how the game works*

	&thkp	requests help with Pepper	*when the child clearly turns to peers and explicitly requests help verbally or nonverbally about how to respond to Pepper*
	&bks	seeks confirmation regarding game	*when the child already has a suggestion for a card but seeks confirmation from peers before making the selection*
	&bkp	seeks confirmation regarding dialogue with Pepper	*when the child already has an answer for Pepper but seeks requests confirmation from peers before saying the answer*
	&skk	entertains or shows off	*when the child shows off to his/her peers (e.g., very exaggerated gestures or facial expressions, throwing him- /herself at the ground, while gazing at peers)*
**Events or behaviors by other actors than game-playing child**			
**Pepper's troublesome behavior**	?pa	Pepper interrupts	*Pepper says something when the child is speaking*
	?pfi	Pepper freezes	*Pepper should behave according to script but does not*
	?pbk	Pepper acts strangely	*Pepper does something asocial or unexpected (e.g., turns to face the corner of the room)*
	?pkk	Pepper make strange comment	*Pepper makes comments that do not fit the context, causing surprise/confusion*
	?psi	Pepper does not respond to the child's answer/question/comment	*Pepper does not react to the child's statement (is prepared and listening, but does not hear/perceive)*
**Interaction from adults**	<fhis	receives help with the game	*when the child is instructed on how the game works without requesting this help*
	<fhip	receives help with Pepper	*when the child is instructed on how to respond to Pepper without requesting this help*
	<isv	adults respond to Pepper	*when the adults speak directly to Pepper, not via the child*

**Interaction from peers**	!fhks	receives help with the game	*when peers make spontaneous suggestions on the game, typically regarding what cards to play*
	!fhkp	receives help with Pepper	*when peers make spontaneous suggestions on possible responses to Pepper*
	!ks	peers disrupt	*when peers talk about off-task topics, engages in internal dialogue, or provide "unwanted" suggestions*
	!kksp	peers respond to Pepper	*when peers speak directly to Pepper, not via the child*
